# Anti-Cancer Activity Profiling of Chemotherapeutic Agents in 3D Co-Cultures of Pancreatic Tumor Spheroids with Cancer-Associated Fibroblasts and Macrophages

**DOI:** 10.3390/cancers13235955

**Published:** 2021-11-26

**Authors:** So-Dam Jang, Jeeyeun Song, Hyun-Ah Kim, Chang-Nim Im, Iftikhar Ali Khawar, Jong Kook Park, Hyo-Jeong Kuh

**Affiliations:** 1 Department of Biomedicine & Health Sciences, Graduate School, The Catholic University of Korea, Seoul 06591, Korea; ujmmm4@catholic.ac.kr (S.-D.J.); jeeyeun2356@catholic.ac.kr (J.S.); yebby1001@catholic.ac.kr (H.-A.K.); 2 Graduate Program for Future Medical Research Leaders, College of Medicine, The Catholic University of Korea, Seoul 06591, Korea; milybud@gmail.com (C.-N.I.); Khawar@wustl.edu (I.A.K.); 3 Department of Biomedical Science, Research Institute for Bioscience & Biotechnology, Hallym University, Chuncheon 24252, Korea; jkpark@hallym.ac.kr; 4 Cancer Evolution Research Center, College of Medicine, The Catholic University of Korea, Seoul 06591, Korea; 5 Department of Medical Life Sciences, College of Medicine, The Catholic University of Korea, Seoul 06591, Korea

**Keywords:** pancreatic cancer, tumor microenvironment, tumor spheroid, 3D culture, anti-cancer drug, pancreatic stellate cell, M2 macrophage

## Abstract

**Simple Summary:**

Pancreatic ductal adenocarcinoma (PDAC) is one of the most aggressive cancers, with a five-year survival rate of less than 8%. There is a need to develop drugs with anti-invasive activity and in vitro tumor models for effective drug screening to improve patient outcomes. Since PDAC invasiveness is mainly induced by tumor-associated stromal cells, we aimed to develop a three-dimensional (3D) PDAC tumor model that mimics in vivo conditions. Additionally, we examined the usefulness of this model for evaluating chemotherapeutic drugs. We succeeded in establishing a 3D co-culture model of multicellular PDAC tumor spheroids, cancer-associated fibroblasts, and tumor-associated macrophages using a microfluidic channel chip platform. We also demonstrated the suitability of this model for evaluating cell-type dependent cytotoxicity, anti-invasive activity, and the association between the two. These results may help develop a novel system for screening the efficacy of chemotherapeutic drugs against PDAC and other solid tumors in the future.

**Abstract:**

Activated pancreatic stellate cells (aPSCs) and M2 macrophages modulate tumor progression and therapeutic efficacy in pancreatic ductal adenocarcinoma (PDAC) via epithelial-mesenchymal transition (EMT). Here, our aim was to analyze the anti-invasion effects of anti-cancer agents where EMT-inducing cancer-stroma interaction occurs under three-dimensional (3D) culture conditions. We used microfluidic channel chips to co-culture pancreatic tumor spheroids (TSs) with aPSCs and THP-1-derived M2 macrophages (M2 THP-1 cells) embedded in type I collagen. Under stromal cell co-culture conditions, PANC-1 TSs displayed elevated expression of EMT-related proteins and increased invasion and migration. When PANC-1 TSs were exposed to gemcitabine, 5-fluorouracil, oxaliplatin, or paclitaxel, 30–50% cells were found unaffected, with no significant changes in the dose-response profiles under stromal cell co-culture conditions. This indicated intrinsic resistance to these drugs and no further induction of drug resistance by stromal cells. Paclitaxel had a significant anti-invasion effect; in contrast, oxaliplatin did not show such effect despite its specific cytotoxicity in M2 THP-1 cells. Overall, our findings demonstrate that the TS-stroma co-culture model of PDAC is useful for activity profiling of anti-cancer agents against cancer and stromal cells, and analyzing the relationship between anti-stromal activity and anti-invasion effects.

## 1. Introduction

Pancreatic ductal adenocarcinoma (PDAC) is one of the deadliest and most aggressive cancers, with poor prognosis and a five-year survival rate of less than 8% [1]. Most patients diagnosed with PDAC present with locally advanced or metastatic disease and are not eligible for surgical resection [2]. As a result, the only standard-of-care treatment option for these patients is chemotherapy, including gemcitabine (GEM), or GEM-based combination regimens, such as GEM/nab-paclitaxel (nab-PTX) or FOLFIRINOX (5-fluorouracil (5-FU), leucovorin, irinotecan, and oxaliplatin (LOHP)). In addition to their limitations in improving overall survival, these treatment options induce drug resistance and exhibit adverse effects on healthy tissues [3]. Studies are ongoing to uncover the mechanisms of PDAC invasiveness and aggressiveness that are related to the tumor microenvironment (TME), and to develop anti-invasive and anti-metastatic drugs.

The epithelial-mesenchymal transition (EMT) of cancer cells is a well-known process that promotes invasion, migration, and drug resistance [4]. Stromal components in the TME play a significant role in inducing EMT in cancer cells. The excessive stromal content in the TME of PDAC is a histopathological hallmark of the disease. The PDAC stroma consists of cellular components such as fibroblasts, pancreatic stellate cells (PSCs), immune cells, and blood vessels, and extracellular matrix (ECM) as the acellular component [5]. During PDAC progression, PSCs are transformed into myofibroblast-type cells characterized by the increased expression of alpha-smooth muscle actin (α-SMA) [6]. Acting as cancer-associated fibroblasts (CAFs), activated PSCs play tumor-supported roles by secreting factors that induce EMT in cancer cells and via ECM deposition and remodeling [7]. CAFs have also recently been shown to have tumor-suppressive roles, which was attributed to their plasticity and heterogeneity [8]. Nevertheless, the contribution of CAFs to tumor progression and therapeutic resistance is well documented, and therefore, they are considered one of the therapeutic targets in cancer. Macrophages are another dominant stromal cell population in the PDAC TME that affects malignant cancer behavior. Peripheral monocytes recruited into the TME differentiate into macrophages and further polarize toward M2 phenotypes, which exert pro-tumor effects by secreting various cytokines and growth factors [9]. These activated PSCs and M2 macrophages, often called tumor(cancer)-associated fibroblasts (TAF/CAFs) and tumor-associated macrophages (TAMs), are major players in tumor progression and poor drug response in PDAC [5]. Moreover, these stromal cells are major targets for anti-stromal therapy development [10].

Recent studies have provided intriguing evidence for therapy-induced stromal activation and its implication in overall anti-cancer efficacy [11]. Stromal cells can modulate the chemotherapy response of cancer cells after exposure to cytotoxic drugs. Furthermore, chemotherapeutic agents such as FOLFOX, cisplatin, taxol, or mitoxantrone damage the stromal cells; however, the altered activity of stromal cells promotes cancer cell survival via cancer-stroma reciprocal interaction [12,13,14,15]. The activity of anti-cancer drugs can be modulated by stromal cells, which can regulate drug metabolism and disposition within tumor tissues. For example, TAM-mediated drug resistance is induced via upregulated drug metabolism, which reduces the active drug concentration within tumors [16]. Hence, targeting stromal components in the TME is a potential therapeutic strategy for improving cancer therapy outcomes [10]. However, there are limited data on the stroma-mediated failure of chemotherapy and its underlying mechanisms [17].

The activity of chemotherapeutic agents should be evaluated in environments that mimic real tumors. Three-dimensional (3D) culture models can recreate the in vivo tumor conditions [18,19]. This is achieved by growing cancer cells as cancer spheroids in a 3D architecture, wherein cell-cell and cell-ECM adhesion and interactions are facilitated [20]. This system allows replication of cellular complexity observed in the TME to study tumor-stroma interplay and its effects [21]. In this study, we established a 3D culture model of PDAC tumors by co-culturing pancreatic tumor spheroids (TSs) with activated pancreatic stellate cells (aPSCs) and THP-1-derived M2 macrophages (M2 THP-1 cells) in a microfluidic channel chip. In addition, we assessed the differential cytotoxicity, as well as anti-invasive and anti-migratory activities of four drugs, GEM, 5-FU, LOHP, and PTX. Furthermore, we found evidence of reciprocal activation between cancer and stromal cells using our 3D co-culture model. Our findings support the notion that the anti-stromal effect of chemotherapeutic agents may not necessarily result in anti-invasive efficacy. This should be considered in the development of anti-stromal therapy.

## 2. Materials and Methods

### 2.1. Cell Culture

PANC-1 (CRL-1469) and BxPC-3 (CRL-1687), human pancreatic cancer cell lines, and THP-1 (TIB-202), a human monocytic cell line, were purchased from the American Type Culture Collection (ATCC; Manassas, VA, USA). Capan-1 (KCLB30079), a human pancreatic cancer cell line, was purchased from the Korean Cell Line Bank (KCLB; Seoul, Korea). Human pancreatic stellate cells (PSCs) were purchased from ScienCell (HPaSteC, #3830, Carlsbad, CA, USA). PANC-1 cells were cultured in high-glucose DMEM (Hyclone, Logan, UT, USA) and BxPC-3, Capan-1, and THP-1 in RPMI1640 (Gibco, Grand Island, NY, USA). Both media were supplemented with 10% fetal bovine serum (FBS; Welgene, Daegu, Korea), 100 μg/mL streptomycin, 100 units/mL penicillin, and 250 ng/mL amphotericin B (Welgene, Daegu, Korea). PSCs were cultured in Stellate Cell Medium (SteCM; #5301, ScienCell, Carlsbad, CA, USA) supplemented with 2% fetal bovine serum, growth supplement, and 1% antibiotic solution, as recommended by ScienCell. The cells were incubated at 37 °C in a humidified atmosphere with 5% CO_2_.

For activation of naive PSC to a myofibroblast-like phenotype, PSCs were cultured in high-glucose DMEM supplemented with 5% fetal bovine serum for at least 72 h before 3D culture (Figure S1A) [22,23]. To induce differentiation and polarization of THP-1-derived M0 and M2 macrophages, THP-1 cells were treated with phorbol-myristate acetate (PMA; P8139, Sigma-Aldrich, St. Louis, MO, USA) at a concentration of 50 ng/mL for 48 h (M0 THP-1 cells), followed by interluekin-4 (IL-4; 200-04, Peprotech, Cranbury, NJ, USA) treatment of 20 ng/mL for 48 h (M2 THP-1 cells) in RPMI 1640 complete medium (Figure S1B).

### 2.2. Preparation of Mirochannel Chip

The microchannel chip design and fabrication methods were performed as previously reported [24]. Briefly, an SU-8 patterned master was prepared using photolithography (AMED, Seoul, Korea), and polydimethylsiloxane (PDMS; SYLGARD 184 Silicone Elastomer Kit, Dow Chemical, Midland, MI, USA) was fabricated using soft lithography procedures. The PDMS solution was prepared by mixing the silicone elastomer base and curing agent in a 10:1 weight ratio and poured onto the patterned master. The PDMS solution was degassed in a vacuum desiccator for 20 min to remove air bubbles, and then cured in a drying oven for 3 h at 60 °C. After curing, the PDMS mold was detached from the master and punched to create media reservoirs and cell loading ports using a biopsy punch and blunt needle. The PDMS mold was autoclaved twice at 120 °C and dried overnight in a drying oven. The PDMS mold was bonded with a glass coverslip after oxygen plasma treatment (CUTE; Femto Science, Seoul, Korea) and immediately treated with poly-dopamine solution (2 mg/mL) to coat the surface of the channel for 2 h, followed by washing twice with deionized water. The microchannel chip was dried in a 60 °C oven for ready use.

### 2.3. Three-Dimensional Co-Culture in Microchannel Chip

**Figure 1:**
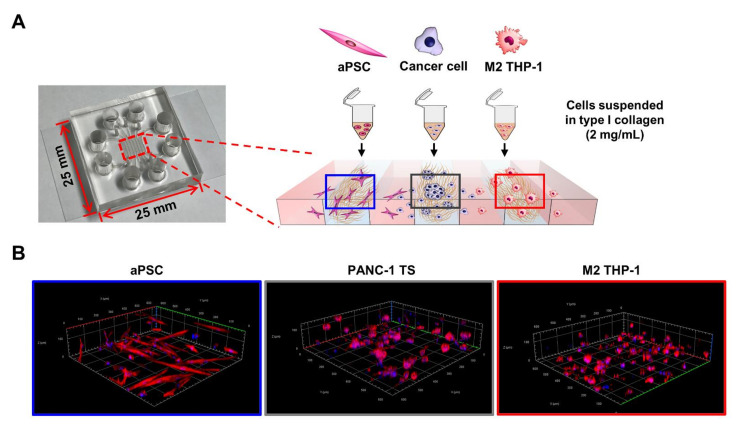
Schematic of the 3D co-culture system using a microfluidic channel chip. (**A**) Cells were pre-conditioned and suspended in type I collagen solution (2 mg/mL). Cells were then loaded into each designated cell channel of the microfluidic channel chip. (**B**) 3D reconstruction images of PANC-1 TSs, aPSCs, and M2 THP-1 cells cultured in collagen matrix. See Materials and Methods for details. TS: tumor spheroid; aPSC: activated pancreatic stellate cell; M2 THP-1: THP-1-derived M2 macrophage.

Cells were suspended at 7 × 10^5^ cells/mL for PANC-1 cells and 1 × 10^6^ cells/mL for BxPC-3 and Capan-1 cells in 2 mg/mL type I collagen solution prepared using phenol red, 0.5 N NaOH, rat tail tendon type I collagen (354236, Corning, Bedford, MA, USA), and sterilized distilled water. PSCs were loaded at the density identical to cancer cells whereas THP-1 cells at two-fold higher density. By injecting 3 μL of the cell-collagen mixture, cells were loaded into each designated channel at 2.1 × 10^3^ cells/channel for PANC-1 cells and 3 × 10^3^ cells/channel for BxPC-3 and Capan-1 cells in the effective area of the channel. For co-culture with stromal cells, cancer cells were loaded into the middle channel and each stromal cell in one of the side channels (Figure 1). The empty channels were filled with cell-free collagen solution. After polymerization in a cell culture incubator with a humidified chamber for 30 min, the microchannels were filled with culture medium and cultured for 5 days in a 5% CO_2_ incubator. For experiments of naive PSC activation by PANC-1 TSs in 3D conditions, SteCM supplemented with 2% FBS were used. In the other experiments which used activated PSCs, high-glucose DMEM with 5% FBS were used. The cell culture medium was changed every two days.

### 2.4. Immunofluorescence Staining

Cells cultured in the microchannel chip were fixed using 4% paraformaldehyde (PFA) for 20 min and permeabilized with 0.1% Triton X-100 for 30 min. Non-specific binding was blocked using 10% normal goat serum for 2 h at room temperature or overnight at 4 °C. The cells were incubated with primary antibodies against α-SMA (1:100, ab5694, Abcam, Cambridge, UK), CD68 (1:50, ab955, Abcam), CTGF (1:100, ab5097, Abcam), cytokeratin 19 (1:200, ab52625, Abcam), E-cadherin (1:200, 3195S, Cell Signaling Technology, Danvers, MK, USA), mannose receptor I (CD206; 1:200, ab125028, Abcam), TGF-β1 (1:200, ab92486, Abcam), and vimentin (1:100, ab92547, Abcam) overnight or for 2 days at 4 °C. Thereafter, the cells were washed with phosphate-buffered saline (PBS). Secondary antibodies, Alexa Fluor anti-rabbit 594 (A11012) or 488 (A11008) and Alexa Fluor anti-mouse 488 (A11029) were used for fluorescence labeling. Rhodamine phalloidin (1:1000, R415, Invitrogen, Carlsbad, CA, USA) and DAPI (1:1000, D9564, Sigma Aldrich, St. Louis, MO, USA) were used to stain F-actin and nuclei, respectively. Images were acquired using a confocal microscope (LSM 800 W/Airyscan, Carl Zeiss, Oberkochen, Germany).

### 2.5. Cell Migration Analysis

To measure migration, cancer cells were cultured alone or co-cultured with aPSCs and M2 THP-1 cells for 5 days. To distinguish cancer cells from aPSCs and M2 THP-1 cells, cytokeratin 19 (1:200, ab52625, Abcam) was stained according to the immunofluorescence staining method. All cell types were observed by staining with rhodamine phalloidin (1:1000, R415, Invitrogen) to stain F-actin. Images were acquired using a confocal microscope, and the number and distance of cancer cells moving from the cell channel to the media channel were analyzed using ImageJ software (National Institutes of Health, Bethesda, MD, USA).

### 2.6. Cell Viability and Invasion Analysis

Cells were treated with anti-cancer drugs on day 3 for 72 h under co-culture conditions based on the microchannel chip. Gemcitabine (GEM), 5-fluorouracil (5-FU), and oxaliplatin (LOHP) were added at concentrations of 0, 0.1, 1, 10, and 100 μM and paclitaxel (PTX) at concentrations of 0, 0.01, 0.03, 0.1, 1, and 10 μM. To measure cell viability, cells were stained with 5 μM calcein AM (BDA-1000, BIOMAX, Seoul, Korea) diluted in culture medium to stain live cells, and then cell viability was measured by calcein AM intensity. Dose-response relationships were determined using an *E_max_* model:

$$\%\;\mathrm{Cell}\;\mathrm{viability}=\left(100-R\right)\times \left(1-\frac{{\left[D\right]}^{m}}{K_{d}^{m}+{\left[D\right]}^{m}}\right)+R$$

where (*D*) is the drug concentration, *K_d_* is the concentration of drug that produces a 50% reduction of the maximum inhibition rate (*E_max_*), m is a Hill-type coefficient and *R* is the residual unaffected (resistance) fraction (*R* = 100 − *E_max_*). IC_50_ was defined as the drug concentration required to reduce viability to 50% of the control (i.e., *K_d_* = IC_50_ when *R* = 0). The curves were fitted using GraphPad Prism 9.0. (GraphPad Software, Inc., San Diego, CA, USA). For measurement of invasion, cells were stained with rhodamine phalloidin (1:1000, R415, Invitrogen) and DAPI (1:1000, D9564, Sigma Aldrich) to stain F-actin and nuclei, respectively. Stained cells were observed using a confocal microscope, and images were analyzed.

### 2.7. Image Acquisition and Analysis

Images were acquired using a confocal microscope. Optical sections were acquired at 6 μm intervals at 50× and 100×, 3 μm at 200×, 2 μm at 400× magnifications and stacked into z-projection images. 3D reconstruction was performed using ZEN software (Carl Zeiss) (Figure 1B). Images for intensity measurement were obtained using z-stack and tile imaging techniques to cover approximately 85% of the effective area in a channel (covered area: 700 × 2400 μm, total area: 700 × 2820 μm). Intensity was determined using ZEN software and normalized to DAPI intensity. To measure the morphological changes of objects, images were acquired at 200× magnification and at least 25 objects per chip were analyzed. For size measurement, three images per chip were acquired at 100× magnification and analyzed. These were analyzed using ImageJ software. The analysis was performed based on previous studies with some modifications [24,25]. The morphology of the TSs was expressed using the shape index defined as perimeter^2^/(4π × area). The diameter of the total objects was calculated as 2 × (area/π)^1/2^. Cells 10–20 μm in diameter were considered as single cells.

### 2.8. Statistical Analysis

All data presented in this study are expressed as the mean ± standard deviation (SD) of three independent experiments. Statistical significance was determined using Student’s *t*-test and one-way ANOVA, followed by Tukey’s post-hoc test using Microsoft Excel 2010 (Microsoft Corporation, Redmond, WA, USA) and GraphPad Prism 9.0. Statistical significance was set at *p* < 0.05.

## 3. Results

### 3.1. Activation and Differentiation of Naive PSCs and THP-1 Cells under PANC-1 TS Co-Culture Conditions

**Figure 2:**
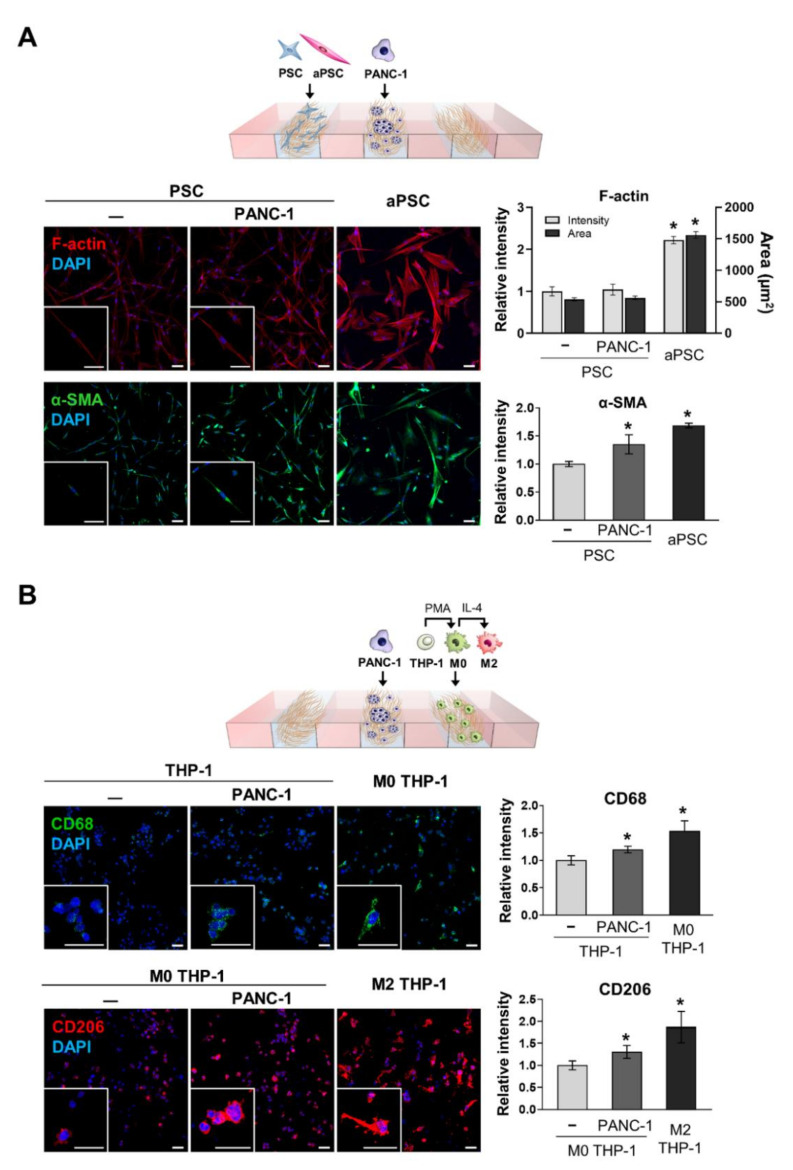
Activation of naive PSCs and THP-1 cells after 3D co-culture with PANC-1 TSs. (**A**) Changes in cellular morphology and the expression level of α-SMA in PSCs under PANC-1 TS co-culture conditions. The effect of TSs on PSC activation was examined by comparing α-SMA expression levels in naive PSCs co-cultured with PANC-1 TSs with those in pre-conditioned aPSCs (See Materials and Methods for details). Green: α-SMA, red: F-actin, blue: DAPI (**B**) Changes in the expression of CD68 (pan-macrophage marker) and CD206 (M2 macrophage marker) in THP-1 cells and M0 THP-1 cells under PANC-1 TS co-culture conditions. The effect of TSs on THP-1 cells polarization was examined by comparing CD68 and CD206 expression levels in THP-1 cells co-cultured with PANC-1 TSs with those of pre-conditioned THP-1 cells (See Materials and Methods for details). Green: CD68, red: CD206, blue: DAPI. Data represent the mean ± SD of at least three independent experiments. Scale bars: 50 µm, * *p* < 0.05 compared to the negative control group. Cells were grown for 5 days before analysis.

The activation of naive PSCs and differentiation and polarization of THP-1 cells were evaluated under PANC-1 TS co-culture conditions for 5 days. Differences in the expression of F-actin and α-SMA were compared between naive PSCs and pre-conditioned aPSCs maintained in high-glucose DMEM supplemented with 5% FBS. aPSCs showed 2.22-fold and 1.68-fold increased expression of F-actin and α-SMA, respectively, compared to control naive PSCs (Figure 2A). Naive PSCs co-cultured with PANC-1 TSs showed 1.35-fold increased expression of α-SMA, approaching 80% of the level observed in pre-conditioned aPSCs. In contrast, no significant change was observed in the expression of F-actin in naive PSCs after co-culture with PANC-1 TSs.

After 5 days of PANC-1 TS co-culture, the expression of CD68 in THP-1 cells and CD206 in M0 macrophages was compared to that in THP-1 cells pre-conditioned to M0 and M2 macrophages, respectively. THP-1 cells showed 1.20-fold higher CD68 expression in the PANC-1 TS co-culture (Figure 2B), which corresponded to 78% of the level observed in THP-1 cells pre-conditioned to M0 macrophages using PMA. When co-cultured with PNAC-1 TSs, M0 THP-1 cells (THP-1-derived M0 macrophages) showed 1.31-fold higher CD206 expression (Figure 2B), reaching 70% of the level in THP-1 cells pre-conditioned to M2 macrophages using PMA and IL-4. These findings indicate that the cellular interactions occurring in our 3D co-culture system were sufficient to induce the activation of naive PSCs and the differentiation of THP-1 cells. To model the interaction between tumor cells and activated stromal cells, pre-conditioned aPSCs and M2 THP-1 cells were loaded onto the 3D co-culture for subsequent experiments.

### 3.2. The Expression of EMT-Related Proteins in Pancreatic TSs Is Increased under Co-Culture with aPSCs and M2 THP-1 Cells

**Figure 3:**
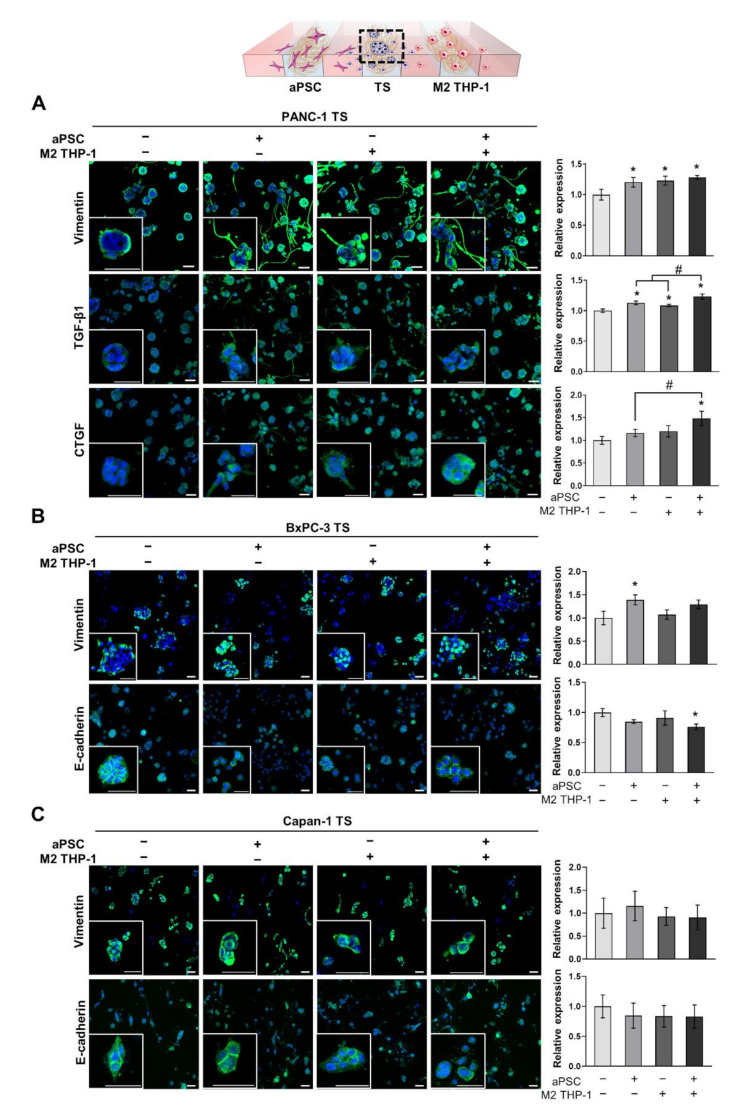
Increased expression of EMT-related proteins in pancreatic TSs under 3D co-culture conditions with stromal cells. (**A**) Expression level of vimentin, TGF-β1, and CTGF in PANC-1 TSs with or without aPSCs, M2 THP-1 cells, or both. (**B**,**C**) Expression level of vimentin and E-cadherin, which are representative EMT markers, in BxPC-3 and Capan-1 TSs with or without aPSCs, M2 THP-1 cells, or both. Cells were counterstained with DAPI. aPSCs and M2 THP-1 cells were pre-conditioned before loading into the microfluidic channels. Data represent the mean ± SD of three independent experiments. Scale bar: 50 µm, * *p* < 0.05 compared to TSs cultured alone. # *p* < 0.05 compared to TSs co-cultured with either aPSCs or M2 THP-1 cells. Cells were grown for 5 days before analysis.

PANC-1, BxPC-3, and Capan-1 TSs were cultured for 5 days with either aPSCs, M2 THP-1 cells, or both. Then, the changes in the expression of EMT-related proteins were evaluated. When co-cultured with either aPSCs, M2 THP-1 cells, or both, the expression of vimentin in PANC-1 TSs was 1.20-, 1.23-, and 1.28-fold higher, respectively, than that in TSs cultured alone (Figure 3A). The acquisition of invasive phenotypes, such as the formation of membrane protrusion, was distinctly observed in PANC-1 TSs under all three stromal cell co-culture conditions. The expression of TGF-β1 showed a similar pattern, with 1.13-, 1.08-, and 1.23-fold higher expression in TSs co-cultured with either aPSCs, M2 THP-1 cells, or both, respectively, than that in TSs alone. In contrast, the expression of CTGF increased by 1.48-fold only when TSs were co-cultured with both stromal cell types. When the effect of either type of stromal cell was compared to that of the combination group, no synergistic effect was observed in the induction of expression for any of three proteins. However, the increases in TGF-β1 and CTGF levels observed in the combination group were statistically significant (Figure 3A).

We also examined the expression of EMT markers, vimentin and E-cadherin, in BxPC-3 and Capan-1 TSs. In contrast to PANC-1 TSs, BxPC-3 and Capan-1 TSs did not show significant changes in the expression of the EMT markers, except for vimentin and E-cadherin in BxPC-3 TSs co-cultured with aPSCs and with both aPSCs and M2 THP-1 cells, respectively (Figure 3B,C). Collectively, these results indicate that stromal cells, such as aPSCs and M2 THP-1 cells, exerted EMT-promoting effects in our 3D culture model; however, these effects occurred in a cell line-dependent manner. Based on the prominent EMT-promoting effect of aPSCs and M2 THP-1 cells in PANC-1 TSs, we selected PANC-1 for further experiments.

### 3.3. aPSCs and M2 THP-1 Cells Increase the Invasion and Migration of PANC-1 Cells

**Figure 4:**
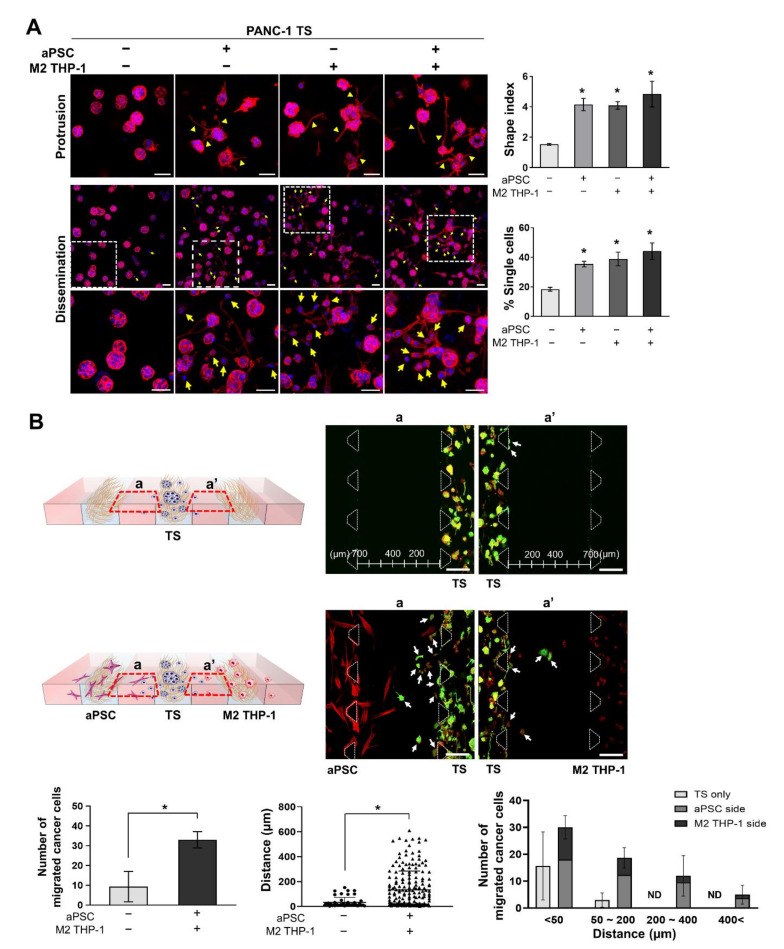
Enhanced invasion and migration capacity of PANC-1 TSs under 3D co-culture conditions with stromal cells. (**A**) Morphological changes in PANC-1 TSs and single cell dissemination under co-culture conditions with aPSCs, M2 THP-1 cells, or both. Red: F-actin, blue: DAPI. Yellow arrow heads indicate membrane protrusions. The morphological changes were expressed using the shape index defined as perimeter^2^/(4π × area). Cells with a diameter between 10 to 20 µm were counted as single cells (yellow arrows). Scale bars: 50 µm. (**B**) The number of cancer cells that moved from the cell channel to the media channel, and the migrated distance were compared between the TS only and co-cultured groups. PANC-1 cells (green) migrated, and those found outside the cell channels are indicated by white arrows. The left graph shows the average number of migrated cancer cells. The graphs in the middle and right show the migrated distance and distribution of the number of migrated cancer cells against distance, respectively. Region (a) and (a′) indicate cells migrated into the media channels from each cell channels. Green: cytokeratin 19, red: F-actin. Data represent the mean ± SD of three independent experiments. Scale bars: 200 µm. * *p* < 0.05 compared to the TS only group. Cells were grown for 5 days before analysis. ND: not detected.

To determine the stromal effect on cancer cell invasion into the ECM and the migration out of the cell channel toward stromal cells, the invasive phenotype was analyzed after 5 days of co-culture. Under stromal cell co-culture conditions, PANC-1 TSs exhibited morphological changes, as indicated by >2-fold increase in shape index caused by membrane protrusion formation (Figure 4A). Many single cells were observed around the membrane protrusions in PANC-1 TSs co-cultured with stromal cells. The number of disseminated single cells increased approximately 2-fold, from 18% to 44.2%. There were no significant differences in shape index and % dissemination between the groups co-cultured with aPSCs, M2 THP-1 cells, or both.

Migratory cancer cells were observed in the media channel when the disseminated single cancer cells moved out of the cell channel. Stromal cells also migrated out of the stromal cell channel and were observed near the cancer cells in the media channel. The number of migratory cells in the media channel increased from nine to 33 per chip, and the distance moved increased from 29.2 μm to 134.4 μm, which correspond to 3.7-fold and 4.6-fold increases, respectively, when comparing PANC-1 TSs cultured alone with those cultured with aPSCs and M2 THP-1 cells (Figure 4B). A significantly higher number of cells moved a distance greater than 50 μm in the co-cultured groups. Furthermore, the cell number on the aPSC side was more than 2-fold larger than that on the M2 THP-1 side. These findings indicate that aPSCs had a greater effect in promoting cancer cells to acquire a high migratory potential than M2 THP-1 cells. Together with the data shown in Figure 3, our findings indicate that in our 3D co-culture model, aPSCs and M2 THP-1 cells could induce EMT and the corresponding changes in PANC-1 TSs toward more invasive and migratory phenotypes.

### 3.4. Co-Culture with aPSCs and M2 THP-1 Cells Does Not Induce Drug Resistance in PANC-1 TSs

**Figure 5:**
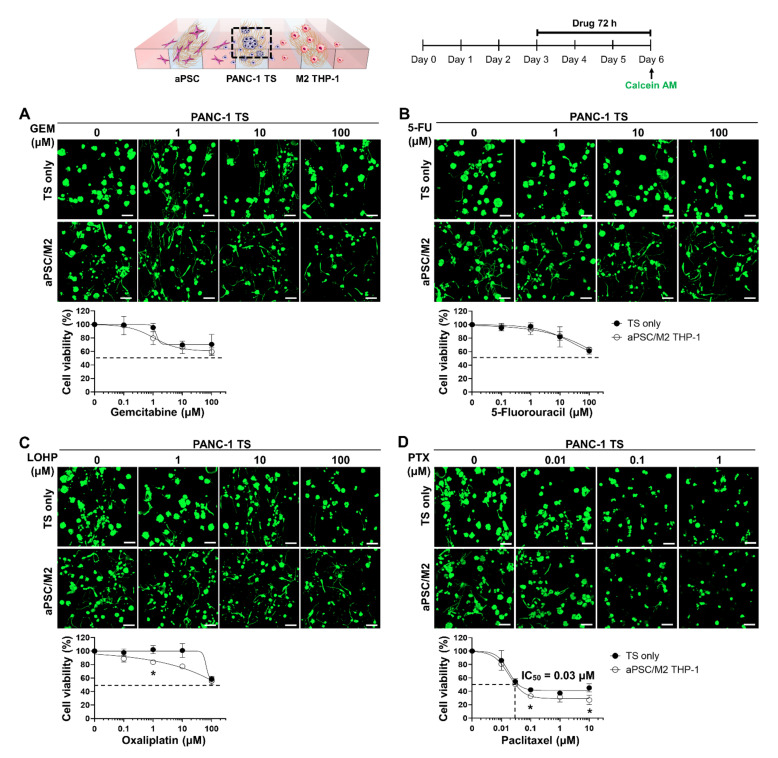
Dose-response curve of the PANC-1 TSs only and co-culture groups when treated with different anti-cancer drugs. Cells grown for 3 days were subjected to 72 h of drug exposure. Cellular viability was measured after calcein AM staining. Representative images and drug-response curves determined in the PANC-1 TS only and co-culture with aPSCs and M2 THP-1 cells are shown for (**A**) GEM, (**B**) 5-FU, (**C**) LOHP, and (**D**) PTX. An IC_50_ value in the cell viability graph of PTX represents the concentration that causes 50% inhibition of cell viability. Data represent the mean ± SD of three independent experiments. Scale bars: 100 μm, * *p* < 0.05 compared to the TS only cultured group. GEM: gemcitabine; 5-FU: 5-fluorouracil; LOHP: oxaliplatin; PTX: paclitaxel.

The effect of tumor-stromal cell interaction on the dose-response relationship was evaluated for four anti-cancer drugs, GEM, 5-FU, LOHP, and PTX, on PANC-1 TSs co-cultured with stromal cells. PANC-1 TSs were cultured with aPSCs and M2 THP-1 cells for 3 days and then exposed to different drugs for 72 h. Except for PTX, which showed an IC_50_ of 0.03 μM and unaffected fraction of less than 30%, treatment with GEM, 5-FU, or LOHP resulted in an unaffected fraction of over 50% at the highest drug concentration tested (100 μM) (Figure 5). For all drugs tested, a decrease in drug sensitivity was not observed under stromal co-culture conditions; instead, a partial increase in the drug response was noted at certain concentrations of LOHP (1 μM) and PTX (0.1 and 10 μM) compared to the TS only group. Collectively, these results indicate that each drug had a unique cytotoxicity profile against PANC-1 TSs, and that co-culture with aPSCs and M2 THP-1 cells did not induce drug resistance.

### 3.5. Different Cytotoxicity Profiles of Anti-Cancer Drugs in Cancer Cells and Stromal Cells

**Figure 6:**
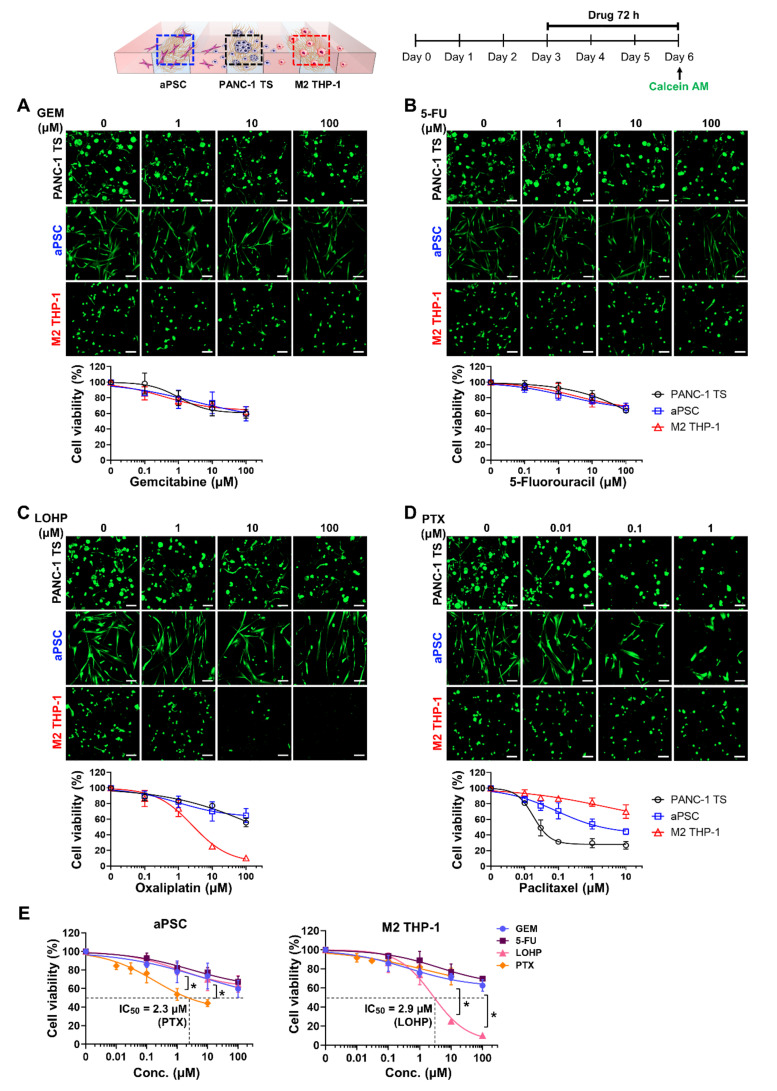
Sensitivity of PANC-1 TSs, aPSCs, and M2 THP-1 cells to anti-cancer drugs under co-culture conditions. Cells grown for 3 days were cultured in drug-containing medium for 72 h. Cellular viability was measured after calcein AM staining. Representative images and drug-response curves of each cell type determined under co-culture conditions, are shown for (**A**) GEM, (**B**) 5-FU, (**C**) LOHP, and (**D**) PTX. Drug-response curve of PANC-1 TSs (**A**–**D**) is the same as that in Figure 5. (**E**) Sensitivity of aPSCs and M2 THP-1 cells to anti-cancer drugs under PANC-1 TSs co-culture conditions. The graphs represent the viability data of aPSCs and M2 THP-1 cells converted from (**A**–**D**). Data represent the mean ± SD of three independent experiments. Scale bars: 100 μm. * *p* < 0.05 compared to the other three drugs.

The dose-response relationships for anti-cancer agents were simultaneously determined in PANC-1 TSs and stromal cells under co-culture conditions. GEM and 5-FU showed similar cytotoxicity in PANC-1 TSs, aPSCs, and M2 THP-1 cells, producing overlapping dose-response profiles (Figure 6A,B). In contrast, LOHP and PTX showed different profiles depending on the cell type. LOHP, which caused comparable cytotoxicity in PANC-1 TSs and aPSCs, showed specific and potent cytotoxicity against M2 THP-1 cells (Figure 6C). When exposed to PTX, each cell type had a unique dose-response profile, with IC_50_ values of 0.03 μM in PANC-1 TSs, 2.3 μM in aPSCs, and over 10 μM in M2 THP-1 cells, showing over 100-fold difference in sensitivity (Figure 6D,E). With respect to stromal toxicity, LOHP and PTX induced prominent cytotoxicity in M2 THP-1 cells (IC_50_, 2.9 μM) and aPSCs (IC_50_, 2.3 μM), respectively, compared to the other agents (Figure 6E).

### 3.6. Comparison of the Anti-Proliferative and Anti-Invasion Effects Induced by LOHP and PTX

**Figure 7:**
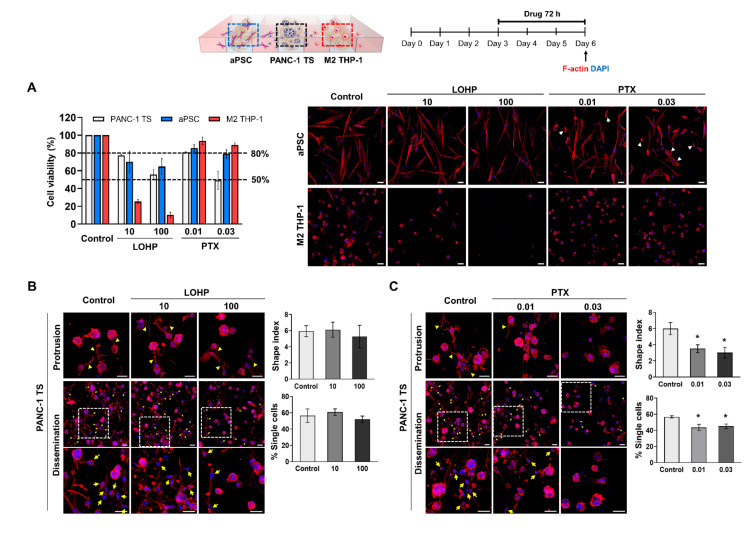
PTX, but not LOHP, exerted anti-invasive effects on PANC-1 TSs under co-culture conditions. Cells grown for 3 days were cultured in drug-containing medium for 72 h. (**A**) Changes in viability of PANC-1 TSs, aPSCs, and M2 THP-1 cells after exposure to LOHP or PTX under co-culture conditions. Red: F-actin, blue: DAPI, white arrow heads indicate the deformed morphology of aPSCs induced by PTX treatment. Cell viability graphs against specific concentrations of LOHP and PTX contain the same data shown in Figure 6C,D. Morphological changes in PANC-1 TSs and single cell dissemination were measured after exposure to (**B**) LOHP and (**C**) PTX. Red: F-actin, blue: DAPI, yellow arrowhead: membrane protrusions, yellow arrow: disseminated single cells. Data represent the mean ± SD of three independent experiments. Scale bar: 50 μm. * *p* < 0.05 compared to control group.

The anti-invasive effects of LOHP and PTX were compared in the concentration ranges that induced approximately 20% to 50% reduction in PANC-1 viability (77% and 56% cell viability observed at 10 μM and 100 μM, respectively, of LOHP; 81% and 50% cell viability at 0.01 μM and 0.03 μM, respectively, of PTX) (Figure 6C,D and Figure 7A). The concentrations at which the two drugs exhibited comparable toxicity in PANC-1 cells, induced notably different toxicities in aPSCs and M2 THP-1 cells (Figure 7A).

There were no changes in the shape index and percentage of disseminated single cells in PANC-1 TSs exposed to LOHP, despite the prominent toxicity caused in M2 THP-1 cells (Figure 7B). To eliminate the residual effect of aPSCs, PANC-1 TSs were exposed to LOHP under co-culture conditions with M2 THP-1 cells, but in the absence of aPSCs (Figure S2). PANC-1 TSs showed no changes in the invasive phenotype under these conditions, indicating that the lack of an anti-invasive effect of LOHP in PANC-1 TSs could not be attributed to the presence of residual aPSCs in the co-culture.

PTX exposure induced an anti-invasive effect in PANC-1 TSs co-cultured with aPSCs and M2 THP-1 cells, as evidenced by a significant decrease in shape index as well as % single-cell dissemination (Figure 7C). Significantly, more than75% aPSCs and M2 THP-1 cells remained viable even when PTX exerted anti-invasive effects, which was a higher survival percentage than that maintained after LOHP exposure (Figure 7A).

## 4. Discussion

In vitro tumor models currently used in cancer research and drug evaluation are highly reductionistic and rarely reflect the complexity of cancer to grant clinical relevance to the data obtained [18]. To recapitulate the in vivo complexity, it is important to include physical, chemical, and biological cues that constitute signaling originating from the tumor and tumor-supporting TME [26]. Such integration can be achieved through the 3D organization of cancer cells and simultaneous co-culture with stromal cells within ECM such as collagen matrix. Organ-on chips and microfluidic technology have made advances in simulating the organs and tissue systems in vitro [27]. Based on these technological advances, we recreated the avascular region of PDAC tumors with a focus on the interplay between tumor cells and tumor-associated stromal cells for drug activity analysis. The present model incorporated two types of stromal cells, aPSCs and M2 macrophages, and allowed indirect tumor-stroma interaction (Figure 1). Similar 3D co-culture models have been developed using different cell types, including cancer cells, fibroblasts, and monocytes within multicellular spheroids formed in U-bottom plates (for pancreatic cancer) [28] or alginate microcapsules (for non-small cell lung cancer) [29]. These have been used to study monocyte recruitment/polarization and the resultant immunosuppressive function, and evaluate the efficacy of therapeutic molecules. Beyond the avascular region tumor model, microfluidic chip-based culture methods have also been utilized for generating tumor models including vascular endothelial cells [30,31]. These have been used for studying tumor angiogenesis and screening drugs for anti-angiogenic activity. Our 3D co-culture model using microfluidic channel chips simulated indirect cancer-stroma interaction and allowed the measurement of cell-type specific changes without additional separation processes. Moreover, our model allows the analysis of 3D spatial and temporal heterogeneity in the culture to track the subpopulations with phenotypic changes [24,32]. Due to low cell number cultured in small size of channel dimension, our model has low suitability for molecular techniques, such as western blotting. Another indirect culture method using membrane chamber (Boyden chamber) has been utilized in cell invasion studies [33]. In heterotypic spheroid models, either cell trackers [34] or fluorescent protein labeling such as GFP and RFP [31] was used for visualizing cells of each type. These methods, however, have limitations due to cytotoxicity or low efficiency and instability of the labeling.

The TME becomes a tumor-supportive environment via malignant transformation of stromal cells into CAFs and TAMs, and contributes to tumorigenesis, metastasis, and chemoresistance [5]. CAFs originate from various cell types, including resident fibroblasts, mesenchymal stem cells, and stellate cells, of which stellate cells are activated into myofibroblasts and act as CAFs in PDAC [7]. Cancer cell-derived factors, Wnt7a via TGF-β-dependent signaling in breast cancer [35] and galectin-3 via integrin beta 1/ILK/NF-κB signaling in pancreatic cancer [36], have been reported to be involved in the malignant transformation of fibroblasts and stellate cells. Other cytokines and factors, such as TGF-β, PDGF, CTGF, IL-10, high glucose (hyperglycemia), and reactive oxygen species (ROS) are also known for stromal transformation [37]. These malignantly transformed CAFs have been considered therapeutic targets because of their tumor-supporting roles [10]. However, tumor-suppressing CAF function have also been identified in two different studies in which targeting CAFs via inhibition of sonic hedgehog signaling [38] and cell lineage specific depletion of α-SMA-positive myofibroblasts [39] exhibited acceleration of tumor growth and reduced survival in PDAC mouse models. This may explain the unexpected failure of IPI-926, an inhibitor of sonic hedgehog signaling, in clinical trials [8]. The opposing roles of CAFs can be attributed to cellular plasticity and phenotypic heterogeneity of CAFs which constitute differential subpopulations such as myofibroblastic and inflammatory CAFs [40,41]. TAMs are another abundant component in the malignant TME of various cancers, including PDAC. Macrophages exhibit plasticity in their phenotypes; the M2-phenotype, rather than the M1-, exhibits pro-tumor properties in cancers [9]. M2 macrophages are polarized by apoptotic cells or factors, such as IL-4, IL-10, IL-13, and TGF-β [42]. GM-CSF and EGF secreted from cancer cells have also been reported to induce M2 polarization of TAMs [43,44]. CAFs recruit monocytes into tumors and promote their polarization toward the M2 phenotype, which facilitates cancer progression via promoting an immunosuppressive microenvironment [45]. In our 3D culture model, PSC activation and M2 polarization were induced under PANC-1 TS co-culture conditions (Figure 2). Although we did not define the factors involved in stromal cell activation in our model, data from our previous publication revealed that PANC-1 TSs secrete various factors, such as CCL-2, EGF, M-CSF, PAI-1, and VEGF [25,46], which are known to induce stromal cell activation [9,37]. In addition, stromal cell activation and function are affected by not only these biochemical factors, but also mechanical cues from the ECM, such as matrix stiffness, topology, and mechanotransduction. Specifically, integrin-mediated interactions in mechanotransduction form focal adhesions in fibroblasts and podosomes in macrophages, leading to cytoskeleton regulation, which affect cellular behaviors such as adhesion, motility, contractility, and phagocytosis [47,48]. These effects may have taken place in our model, where cells were cultured in embedded type I collagen, which is one of the ECM components and may operate with biochemical factors to regulate stromal cell activation.

EMT is a non-cell autonomous process involving paracrine signaling from activated stromal cells, CAFs, and TAMs [4]. Various factors secreted from CAFs or TAMs, such as TGF-β, SDF-1, and CCL18, induce EMT, invasiveness, migration, and stemness in cancer cells, as reported by studies that used conditioned medium stimulation or indirect co-culture in Transwells [49,50,51]. SMAD4 is critical for TGF-β-driven EMT and induces changes in the expression of EMT markers in human pancreatic cancer cells [52,53]. PANC-1 expresses wild-type *SMAD4*, whereas BxPC-3 and Capan-1 harbor a homozygous deletion and point mutation, respectively, in *SMAD4*. Consequently, these cell lines lack the SMAD4 protein [54]. Accordingly, partial EMT is induced in BxPC-3 cells, showing a decrease in E-cadherin expression without mesenchymal marker expression following TGF-β treatment [55]. In Capan-1 cells, no EMT-related alterations in cellular morphology and protein expression are observed [56]. Consistent with these observations, we did not find apparent EMT-induced effects in BxPC-3 and Capan-1 TSs under co-culture conditions with aPSCs and M2 THP-1 cells (Figure 3B,C). On the contrary, EMT was evident in PANC-1 TSs, as demonstrated by the increased expression of EMT-related proteins, such as vimentin, TGF-β1, CTGF, and increased invasion and migration (Figure 3A and Figure 4). In addition, we observed fibronectin remodeling around PANC-1 TSs, including increased fibronectin alignment, thickness, and degradation by invading PANC-1 cancer cells [57] as in our previous reports [24,25].

The role of EMT in the induction of drug resistance has been demonstrated in many studies [58]. When EMT occurs in cancer cells, increased invasion and migration abilities are commonly accompanied by the induction of drug resistance [59,60]; thus, targeting EMT is an effective strategy for the development of anti-metastatic therapy [58]. Several studies have presented data using genetic lineage tracing to oppose the requirement of EMT for metastasis in pancreatic and breast cancers; however, EMT-mediated chemoresistance has not been questioned [61,62,63]. In our study, no significant changes in drug susceptibility were observed when PANC-1 TSs were co-cultured with tumor-supporting stromal cells (Figure 5), whereas the promotion of EMT was evidenced by the increased marker expression and elevated invasive and migratory abilities (Figure 3A and Figure 4). The PANC-1 cells used in the present study exhibit several mesenchymal characteristics among several pancreatic cancer cell lines, with low levels of E-cadherin and high levels of vimentin expression [24,25]. PANC-1 cells show relatively high intrinsic resistance to drugs, such as GEM, cisplatin, and 5-FU compared to other cell lines with an epithelial phenotype [25,64]. The PANC-1 cell population that acquired migratory and invasive phenotypes showing cellular protrusion was accounted for less than 10% of the total population, which increased from 4.9% to 8.4% under the EMT-promoting influence of stromal cells [57]. Additionally, the absence of changes in Ki-67 expression indicated a negligible increase in cell proliferative activity under stromal cell co-culture (Figure S3). Based on these data, we speculate that EMT in PANC-1 cells promoted a matrix-invading phenotype in a small portion of cells but was not sufficient to induce overall chemoresistance in PANC-1 cells, which are already drug-insensitive with mesenchymal properties. This hypothesis needs to be tested using various cell lines with different levels of mesenchymal properties.

LOHP did not show significant anti-invasive activity despite its specific and potent cytotoxicity towards M2 THP-1 cells (Figure 7A,B and Figure S2). The cytotoxicity of LOHP towards M2 THP-1 cells has been reported elsewhere. In fact, the intraperitoneal administration of LOHP was found to decrease the number of TAMs in the abdominal implantation model of colon cancer [65]. This observation is inconsistent with the results shown in Figure 4A, which reveals that co-culture with M2 THP-1 cells significantly induced protrusion formation and dissemination in PANC-1 cells. We speculate that the EMT-inducing effect of M2 THP-1 cells is either irreversible once induced or sustained for long duration; hence, matrix invasion and migration were not promptly abrogated when M2 THP-1 cell viability was compromised. Further studies evaluating the long-term effect of LOHP (i.e., later than 72 h) may differentiate these two possible mechanisms. Another possible mechanism underlying the insignificant anti-invasion effect of LOHP may be associated with its unexpected effect on EMT induction with ROS production, which is mediated through the upregulation of Snail1 via the PI3K-Akt pathway in colon cancer and hepatocellular carcinoma [66,67]. In contrast to LOHP, PTX exerted significant anti-invasive effects, as evidenced by the results of protrusion formation and cell dissemination (Figure 7C). These finding may be attributed to the potent activity of PTX, directly acting on microtubule stabilization and disturbing the motility and invasion of PANC-1 cells. This is because microtubule dynamics plays a pivotal role to provide mechanical support to actin-based protrusions [68,69,70]. In addition, both PTX and nab-PTX induce the reprogramming of M2 macrophages to the M1 phenotype in a TLR4-dependent manner, thus reducing tumor growth [71,72]. The possible contribution of this repolarization effect to the anti-invasive activity of PTX warrants further studies.

## 5. Conclusions

We established a microfluidic channel chip-based PDAC tumor model in which PANC-1 TSs and the tumor-associated stroma cells, aPSCs and M2 macrophages, were co-cultured to allow interaction in a 3D environment. The usefulness of the model was demonstrated through activity profiling of anti-cancer agents in cancer and stromal cells, and the analysis of the relationship between anti-stromal activity and anti-invasion effects. Targeting EMT-inducing stroma for testing anti-invasion efficacy may not be effective when the acquisition of an invasive phenotype in cancer cells is no longer stroma-dependent, which warrants further investigation.

## Data Availability

The data presented in this study are available on request from the corresponding author.

## Supplementary Materials

The following are available online at https://www.mdpi.com/article/10.3390/cancers13235955/s1, Figure S1: Activation of naive PSC and differentiation of THP-1 cells under 2D culture conditions. Figure S2: PANC-1 TSs maintain invasive phenotype despite oxaliplatin treatment under M2 THP-1 cells co-culture conditions. Figure S3: Expression level of Ki-67, a cell proliferation marker, in PANC-1 TSs cultured alone and those co-cultured with aPSCs and M2 THP-1 cells.
