# Supplementary material for: Anti-Cancer Activity Profiling of Chemotherapeutic Agents in 3D Co-Cultures of Pancreatic Tumor Spheroids with Cancer-Associated Fibroblasts and Macrophages
Source: Cancers (Basel). 2021 Nov 26;13(23):5955. doi: 10.3390/cancers13235955 (PMC8656537; doi:10.3390/cancers13235955)
Supplement: Supplementary file 1 [file cancers-13-05955-s001.zip › cancers-1450331-supplementary.pdf]

Supplementary Materials

Anti-Cancer Activity Profiling of Chemotherapeutic Agents in 3D Co-Cultures of Pancreatic Tumor Spheroids with Cancer-Associated Fibroblasts and Macrophages

So-Dam Jang, Jeeyeun Song, Hyun-Ah Kim, Chang-Nim Im, Iftikhar Ali Khawar, Jong Kook Park and Hyo-Jeong Kuh

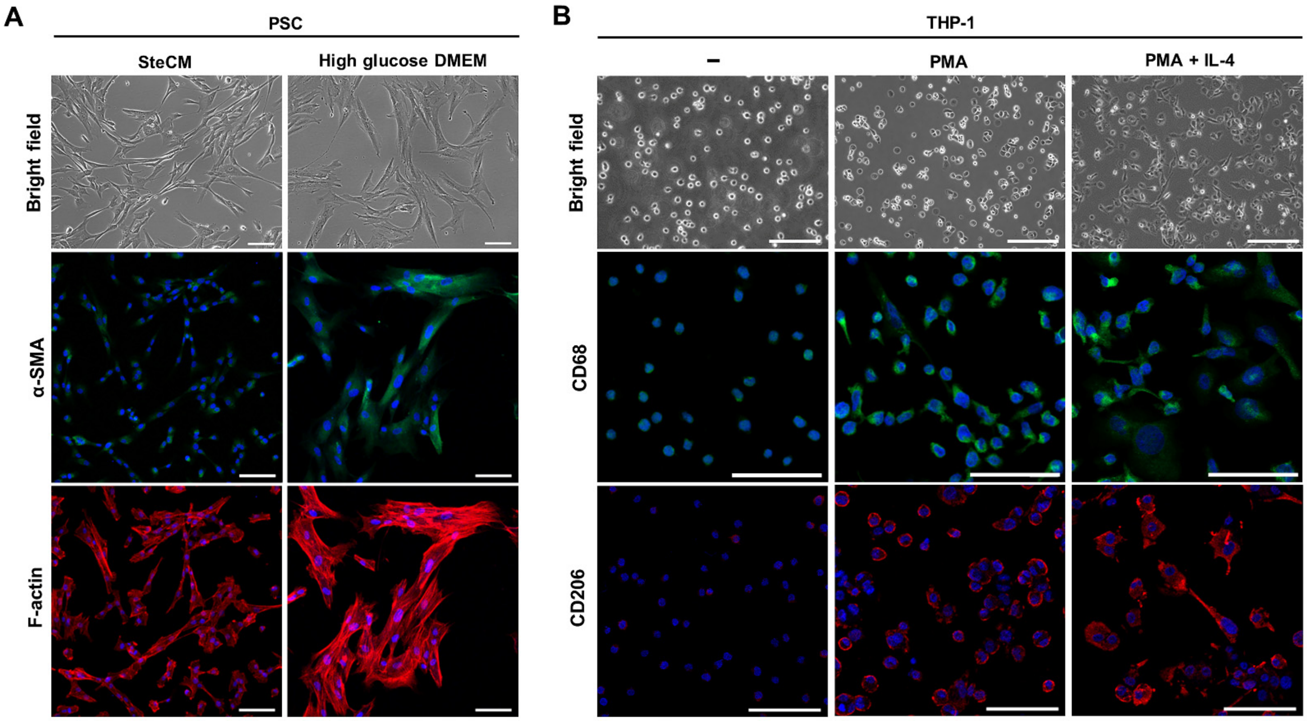

Figure S1. Activation of naive PSCs and differentiation of THP-1 cells under 2D culture conditions. (**A**) Bright field images and confocal images of α-SMA and F-actin expression in naive and activated PSCs. Naive PSCs were maintained using Stellate Cell Medium (SteCM) supplemented with 2% FBS, and activated by culturing in high glucose DMEM supplemented with 5% FBS for at least 72 h. (**B**) Bright field images and confocal images showing CD68 and CD206 expression in THP-1 cells with or without PMA and IL-4 treatment. THP-1 cells were differentiated into M0 macrophages by treating with 50 ng/mL for 48 h, and polarized to M2 macrophages with PMA treatment, followed by the addition of 20 ng/mL IL-4and incubation for 48 h. Scale bar: 100 μm.

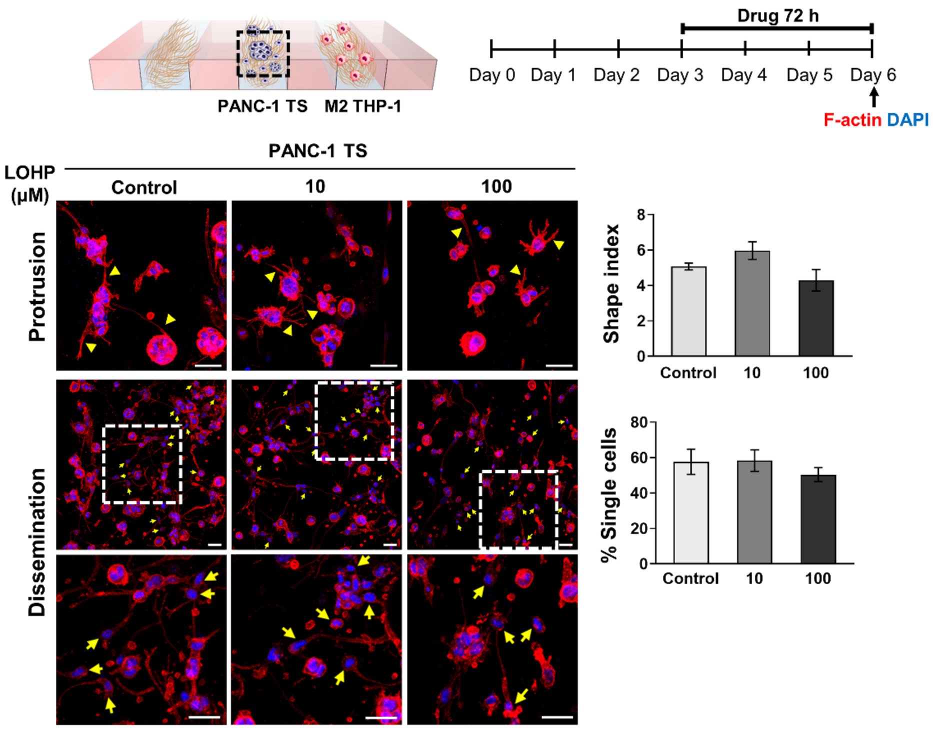

Figure S2. PANC-1 TSs maintain invasive phenotype despite oxaliplatin treatment under M2 THP-1 cell co-culture conditions. Cells grown for 3 days were exposed to LOHP-containing medium for 72 h. The morphology and single cell dissemination of PANC-1 TSs treated with or without drug were compared under M2 THP-1 cell co-culture conditions. Red: F-actin, Blue: DAPI, yellow arrowhead: membrane protrusions, yellow arrow: disseminated single cells. Data represent the mean ± SD of three independent experiments. Scale bar: 50 μm.

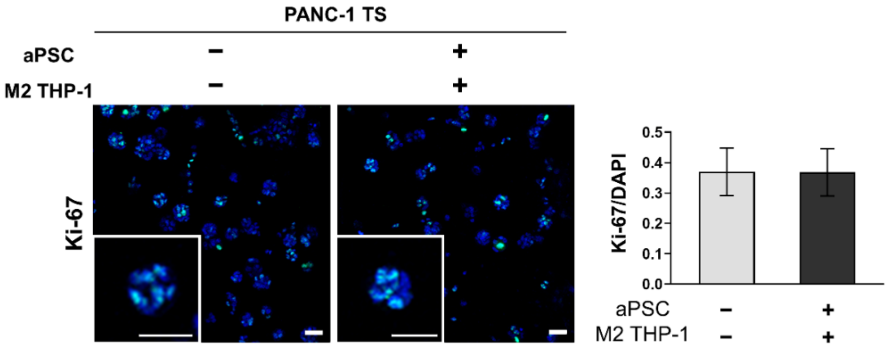

Figure S3. Expression level of Ki-67, a cell proliferation marker, in PANC-1 TSs cultured alone and those co-cultured with aPSCs and M2 THP-1 cells. Green: Ki-67, blue: DAPI. Data represent the mean ± SD of three independent experiments. Scale bar: 50 μm.
